# Trehalose induces bladder smooth muscle hypercontractility in mice: involvement of oxidative stress and cellular senescence

**DOI:** 10.3389/fphys.2025.1572139

**Published:** 2025-04-04

**Authors:** Guilherme Lemos, Cícera Madri Alves de Souza Fernandes, Ingrid Kazue Mizuno Watanabe, Maria Andreia Delbin, Fábio Henrique Silva, Fabiano Beraldi Calmasini

**Affiliations:** ^1^ Department of Pharmacology, Escola Paulista de Medicina, Universidade Federal de São Paulo, São Paulo, Brazil; ^2^ Department of Medicine, Nephrology Division, Universidade Federal de São Paulo, São Paulo, Brazil; ^3^ Departamento de Biologia Estrutural e Funcional, Instituto de Biologia, Universidade Estadual de Campinas (UNICAMP), Campinas, Brazil; ^4^ Laboratory of Multidisciplinary Research, São Francisco University (USF), Sao Paulo, Brazil

**Keywords:** overactive bladder, autophagy, NADPH oxidase, superoxide anion, reactive oxygen species

## Abstract

Autophagy, a conserved catabolic process, is critical for cellular homeostasis and its dysregulation has been implicated in a number of conditions including hypertension, obesity and bladder dysfunctions. The autophagy inducer trehalose has shown promise in treating diseases; however, some studies have reported detrimental effects in vascular tissue under health conditions. In the bladder, the effects of trehalose remain unclear. Therefore, in the present study, male C57BL6/JUnib mice (8 weeks old) were divided into control and trehalose-treated groups (120 mg/mouse/day via gavage) for 4 weeks. After treatment, bladders were harvested for functional, biochemical, and molecular analyses. The trehalose treatment increased the bladder smooth muscle (BSM) contractility to carbachol (CCh), without altering relaxation response to isoproterenol. The CCh-induced BSM hypercontractility was completely abolished by the *in vitro* incubation of apocynin and diphenyleneiodonium (DPI), implicating NADPH oxidase-derived reactive oxygen species (ROS) on this process. Accordingly, increased levels of superoxide anion (O^2-^) were found in the urothelial layer, but not in BSM, of trehalose-treated mice. Trehalose also increased senescence-associated β-galactosidase activity in the bladder but failed to upregulate autophagy-related proteins LAMP1 and Beclin-1 in the bladder. Collectively, we show for the first time that trehalose induces BSM hypercontractility in mice, linked to increased levels of O^2-^ and senescent cell, independently of autophagy activation. Therefore, trehalose administration is an effective model for studying BSM hypercontractility in mice, particularly associated with oxidative stress and cellular senescence.

## Introduction

Autophagy, also referred to as macroautophagy, is a highly conserved catabolic process responsible for the removal of damaged proteins and organelles while supplying nutrients to cells during periods of energy deprivation (Ren and Zhang, 2018). Impairments in the autophagy process have been implicated in the pathophysiology of a number of conditions, including hypertension (McCarthy et al., 2019), obesity (Fernandez et al., 2017) and lower urinary tract symptoms (LUTS; De Nunzio et al., 2017).

Specifically, in the lower urinary tract, the expression and activity of autophagy-related proteins have been demonstrated in the bladder urothelium and smooth muscle in both humans and mice (Erman et al., 2013; Larson et al., 2010). The autophagy activation mitigated bladder remodeling and preserved voiding function in rats with partial bladder outlet obstruction or cyclophosphamide-induced cystitis (Schroder et al., 2013; Zhao et al., 2015), highlighting a critical role of autophagy in the bladder under pathological conditions.

The primary signaling pathway regulating autophagy involves the mammalian target of rapamycin (mTOR) protein, which acts as a negative modulator, playing a key role in suppressing autophagy. Thus, for autophagy to occur, mTOR must be inhibited, either physiologically (e.g., by adenosine monophosphate-activated protein kinase, AMPK) or pharmacologically (e.g., by drugs such as rapamycin) (Wang et al., 2022; Boutouja et al., 2019). Additionally, m-TOR-independent autophagic activation pathways have been explored in the literature, with trehalose emerging as one of the most prominent compounds used (Pupyshev et al., 2022).

Trehalose is a disaccharide consisting of two glucose molecules linked by a stable bond that can only be cleaved by specific enzymes known as trehalases (Elbein et al., 2003). The exact pathway by which trehalose triggers autophagy remains not fully understood. One of the proposed mechanisms involves the inhibition of GLUT transporter on the cell membrane, which blocks the entry of glucose and fructose into the intracellular space, creating a starvation-like state within the cell and activating key regulators of autophagosome formation (Mardones et al., 2016).

In the literature, trehalose has demonstrated efficacy in treating a number of diseases. For instance, trehalose reduced hypertension-induced vascular impairments in spontaneous hypertensive rats (McCarthy et al., 2019) and promoted a neuroprotective effect in a mouse model of Parkinson’s disease (Pradeloux et al., 2024). However, some studies have also reported that, under physiological conditions, trehalose is associated with arterial impairments in rats, characterized by reduced vascular smooth muscle relaxation, increased oxidative stress, and accelerated vascular aging (McCarthy et al., 2019). Nevertheless, the effects of trehalose on the lower urinary tract remain poorly understood. Therefore, this study aimed to investigate the impact of trehalose treatment in mice, with a specific focus on bladder tissue. We hypothesized that trehalose may induce bladder dysfunction by enhancing smooth muscle contractility, promoting ROS production, and leading to cellular senescence.

## Material and methods

### Animals

Eight-week-old male C57BL6/JUnib mice weighing 28 ± 0.4 g at the beginning of the study were provided from Laboratory of Animal Experimentation of the National Institute of Pharmacology and Molecular Biology (LEA-INFAR) - Federal University of Sao Paulo and housed in cages (three mice per cage). Animals were house in temperature-controlled facilities on a 12-h light/dark cycle with *ad libitum* access of water and standard food. All the procedures and protocols were approved by Ethics Committee for the Use of Experimental Animals, Federal University of Sao Paulo (CEUA-UNIFESP; protocol number 1421040323).

### Experimental design

The mice were randomly divided into two groups, namely, control and trehalose, through simple randomization. In the trehalose group, the animals received a 4-week treatment with trehalose (120 mg/mouse/per day, gavage, in tap water). The trehalose dose was estimated based on previous studies (Pupyshev et al., 2019; McCarthy et al., 2019). The control group received only tap water (300 uL/mouse/day, gavage). After the conclusion of the 4-week trehalose treatment, the animals were euthanized by isoflurane overdose and the bladder were removed for the functional, biochemical and molecular assays, as described below.

### *In vitro* functional protocols in isolated bladder

The *in vitro* functional experiments were conducted based on previous studies that investigated bladder contraction and relaxation in mice (Alexandre et al., 2016; Calmasini et al., 2017). The bladder was removed from the animals to a petri-dish containing Krebs-Henseleit solution and carefully divided into two longitudinal strips. The strips were mounted in 5-mL myograph filled with Krebs-solution at 37°C, pH 7.4, composed of 117 mM NaCl, 4.7 mM KCl, 2.5 mM CaCl_2_, 1.2 mM MgSO_4_, 1.2 mM KH_2_PO_4_, 25 mM NaHCO_3_ and 11 mM glucose, and continuously oxygenated with a mixture of 95% O_2_ and 5% CO_2_. The alterations in the isometric force were recorded by a PowerLab system (ADInstruments). The resting tension applied to the tissues (5 mN) was periodically adjusted until stabilization (60 min), and the Krebs solution was replaced every 15 min. Cumulative concentration-response curves to the contractile agent carbachol (a muscarinic receptor agonist; CCh, 1 nM–30 µM) and to the relaxing agent isoproterenol (a beta-adrenoceptor agonist; ISO, 1 nM–10 µM) were conducted in intact bladder from control and trealose-treated mice. In a separated set of experiments, we tested the role of ROS levels in the CCh-induced bladder contractility in control and trehalose groups. Accordingly, intact bladder strips from both groups were incubated or not with apocynin (antioxidant agent, 100 µM) or diphenyleneiodonium (DPI; a selective NADPH oxidase inhibitor, 5 µM) for a period of 30 min and thereafter, concentration-response curves to CCh were performed.

### Quantification of reactive oxygen species (ROS)

These experiments were based on previous studies that investigated ROS levels in the bladders of mice (Alexandre et al., 2016; Oliveira et al., 2022). The oxidative fluorescent dye hydroethidine (dihydroethidium, DHE; Invitrogen, Grand Island, NY, United States) was employed to assess the bladder ROS generation. The bladder was placed in a freezing medium (Tissue-Tek O.C.T. Compound, Sakura, CA, United States), and 12 μm transverse sections of frozen tissue were prepared using a cryostat, mounted on glass slides, and allowed to equilibrate for 10 min in Hanks’ solution (in mM: 1.6 CaCl_2_, 1.0 MgSO_4_, 145 NaCl, 5.0 KCl, 0.5 NaH_2_PO_4_, 10 dextrose, 10 HEPES, pH 7.4) at 37°C. Fresh Hanks’ solution containing DHE (2 μM) was added to each tissue section, which were then incubated for 30 min in a light-protected, humidified chamber at 37°C. Images were captured using a microscope (Eclipse 80i; Nikon, Tokyo, Japan) equipped for epifluorescence (excitation at 488 nm; emission at 610 nm) with a camera (DS-U3; Nikon). Fluorescence was measured using a 585-nm long-pass filter. The number of nuclei stained with ethidium bromide (EB-positive nuclei) throughout the bladder smooth muscle and urothelium was automatically quantified using ImageJ Software (National Institutes of Health, Bethesda, MD, United States) and expressed as labeled nuclei per square millimeter.

### Quantification of senescent cells

The quantification of senescent cells was performed using a commercially available kit (Cell Signaling, Beverly, MA, United States). The bladders from control and trehalose-treated mice were removed and placed in freezing medium (Tissue-Tek O.C.T. Compound, Sakura, CA, United States). The tissues were sectioned (10 μm) using a cryostat and placed on glass slides coated with silane. The slides were washed with PBS and fixed for 15 min at room temperature with the fixative solution provided in the kit. Subsequently, the slides were washed twice with PBS, and a staining solution (β-galactosidase staining solution) was added. The slides were incubated for 12 h in a dry incubator at 37°C in the presence of the staining solution. After this period, the slides were photographed using an optical microscope, and the analysis was performed using the ImageJ image software. The data were expressed as the percentage of the area marked per microscopic field.

### Western blot analysis

To assess the expression of autophagy-related proteins, bladder was removed and homogenized, in RIPA buffer containing inhibitor of proteases, using a Precellys Evolution tissue homogenizer followed by centrifugation at 14,600*g* at 4°C for 20 min to remove insoluble components. The protein concentrations of the supernatants were determined using the Bradford assay, and equal amounts of protein from each sample (30 μg) were treated with Laemmli buffer containing dithiothreitol (100 mM). The samples were heated at 95°C for 10 min and separated by sodium dodecyl sulfate polyacrylamide gel electrophoresis (8% or 15%). Protein transfer to a nitrocellulose membrane was carried out for 45 min at 15 V (constant) in a Trans-Blot Turbo System (Bio-Rad, Hercules, CA, United States). The primary antibodies used were anti-LAMP-1 (sc-7985; 1:200) and anti-Beclin-1 (sc-8312; 1:1,000) from Santa Cruz Biotechnologies (Santa Cruz, Dallas, TX, United States). Detection was performed using horseradish peroxidase-conjugated secondary antibodies and a Luminol solution. Densitometric analysis was carried out using Image software (Scion, Frederick, MD, United States), and the results were expressed as the ratio of protein expression divided by α-actin or GAPDH.

### Statistical analysis

All data are expressed as means ± S.E.M. The GraphPad Prism Program (GraphPad Software Inc.) version 5.0 was used for statistical analysis. The Shapiro-Wilk test was used to assess the normal distribution of the data. Unpaired Student’s t test was used to compared two groups and one-way ANOVA followed by Bonferroni’s tests was used to compare more than two groups. P < 0.05 was accepted as significant.

## Results

### Body weight, bladder weight and glycemia profile

Table 1 shows that trehalose did not affect body weight or fasting blood glucose in treated mice compared with control group. Similarly, bladder weight was not different between control and trehalose-treated group.

**TABLE 1:** Body weight, bladder weight and blood glucose in control and trehalose-treated mice.

	Control group	Trehalose group
Initial body weight (g)	28.3 ± 0.6	28.7 ± 0.4
Final body weight (g)	30.6 ± 0.7	29.8 ± 0.3
Fasting blood glucose (mg/dL)	126.3 ± 7.8	118.2 ± 7.1
Bladder weight (mg)	22.5 ± 0.5	23.4 ± 1.9

### Trehalose treatment increases mouse bladder smooth muscle contractility

**FIGURE 1:**
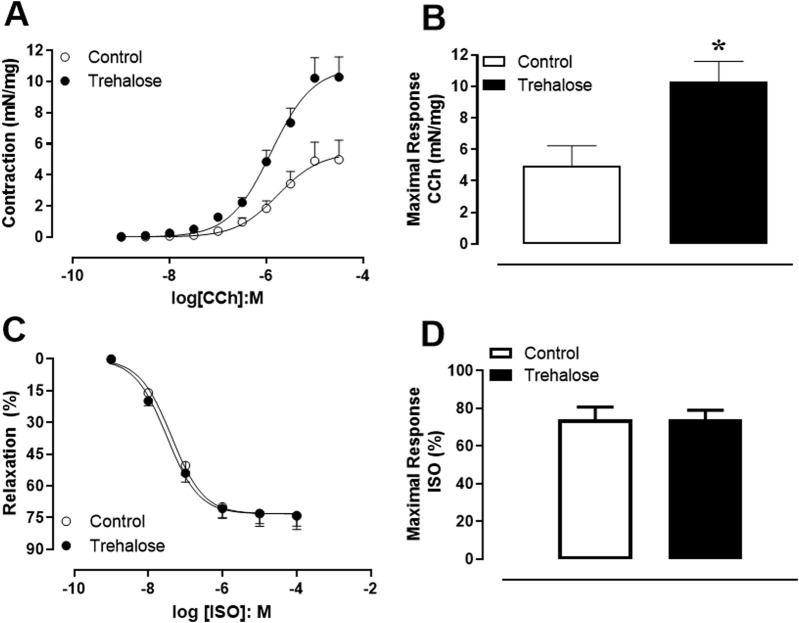
Concentration-response curve to carbachol (CCh; 1 nM–30 µM, **(A)** isoproterenol (ISO; 1 nM–100 µM; **(C)** and maximal response to CCh **(B)** and ISO **(D)** in bladder smooth muscle from control and trehalose-treated mice. Data represent the mean ± S.E.M. (n = 5-9). *P < 0.05 compared with control group.

Concentration-response curves to contractile and relaxing agents were performed in intact bladder strips from control and trehalose-treated mice (Figure 1). Addition of carbachol (CCh, 1 nM–30 µM) elicited concentration-dependent contractions in both groups (Figure 1A); however, in the trehalose-treated group, the maximal responses (Emax) were higher for carbachol (Figure 1B; P < 0.05, Hedges’ *g* = 1.49) compared with control group. On the other hand, addition of isoproterenol (ISO; 1 nM–10 µM) produced concentration-dependent relaxing effect in bladder strips with no differences between groups (Figures 1C, D). The potency (pEC_50_) values were not different between control (5.76 ± 0.17 and 7.37 ± 0.11) and trehalose-treat group (5.90 ± 0.09 and 7.50 ± 0.09) for CCh and ISO, respectively.

### Implication of ROS on trehalose-induced bladder smooth muscle hypercontractility

**FIGURE 2:**
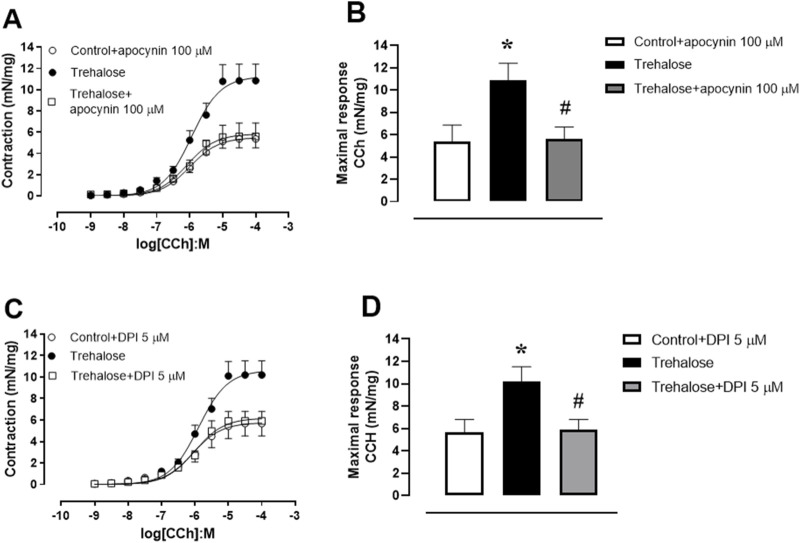
Concentration-response curve to carbachol (CCh; 1 nM–30 µM, **(A, C)** and maximal response **(B, D)** in bladder smooth muscle from control and trehalose-treated mice preincubated with apocynin (100 µM - 30 min; **(A, B)** and diphenyleneiodonium (DPI; 5 µM–30 min, **(C, D)** Data represent the mean ± S.E.M. (n = 6-9). *P < 0.05 compared with control group and ^#^P < 0.05 compared with control + trehalose.

Figures 2A, B shows that prior incubation with the antioxidant agent apocynin (100 μM–30 min) fully restored the bladder smooth muscle hypercontractility to CCh in trehalose-treated group (P < 0.05). Similarly, the incubation with the diphenyleneiodonium, a selective NADPH oxidase inhibitor (DPI, 5 μM–30 min) also restored the bladder smooth muscle hypercontractility to CCh in trehalose-treated group (Figures 2C, D; P < 0.05). No differences were seen in pEC_50_ values for apocynin (control plus apocynin 5.99 ± 0.21, trehalose-treated group: 5.93 ± 0.11 and trehalose-treated group plus apocynin 6.07 ± 0.15; Figure 2A) or DPI (control plus DPI: 6.07 ± 0,17, trehalose-treated group: 5.89 ± 0.10 and trehalose-treated group plus DPI: 6.01 ± 0.12; Figure 2C).

### Levels of ROS in bladder tissue

**FIGURE 3:**
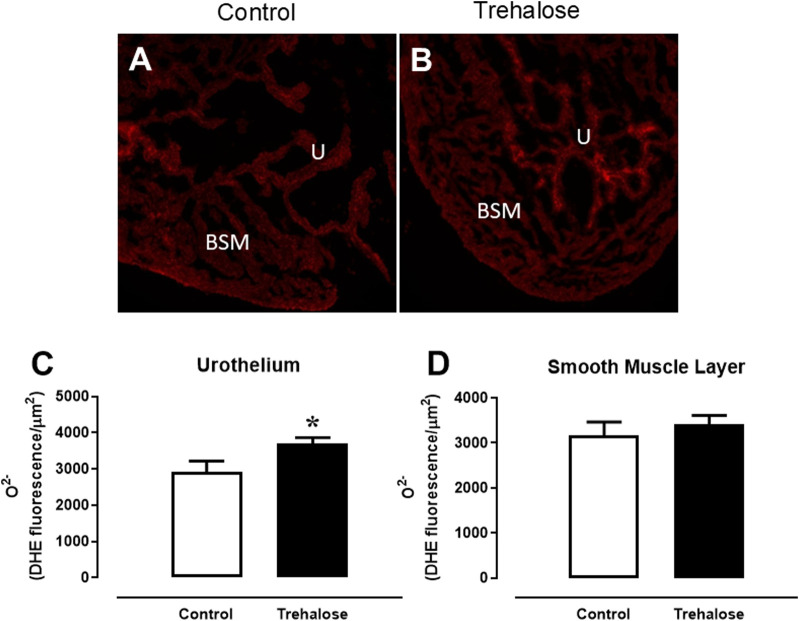
Superoxide anion (O^2-^) levels through DHE-induced fluorescence **(A, B)** and quantification of ethidium-bromide-positive nuclei in the urothelium **(C)** and bladder smooth muscle **(D)** in bladder from control and trehalose-treated mice Data represent the mean ± S.E.M. (n = 6–7). *P < 0.05 compared with control group.

The fluorescent dye dihydroethidium (DHE) was used in fresh-frozen bladder sections of control and trehalose-treated mice (Figure 3). Under identical conditions, the fluorescent intensity was 27.2% higher in the bladder urothelial layer (Figure 3C, P < 0.05, Hedges’ *g* = 1.36) of trehalose-treated group compared with control mice. No difference in fluorescent intensity was observed in bladder smooth muscle layer between the groups (Figure 3D).

### Senescence associated-β-galactosidase staining

**FIGURE 4:**
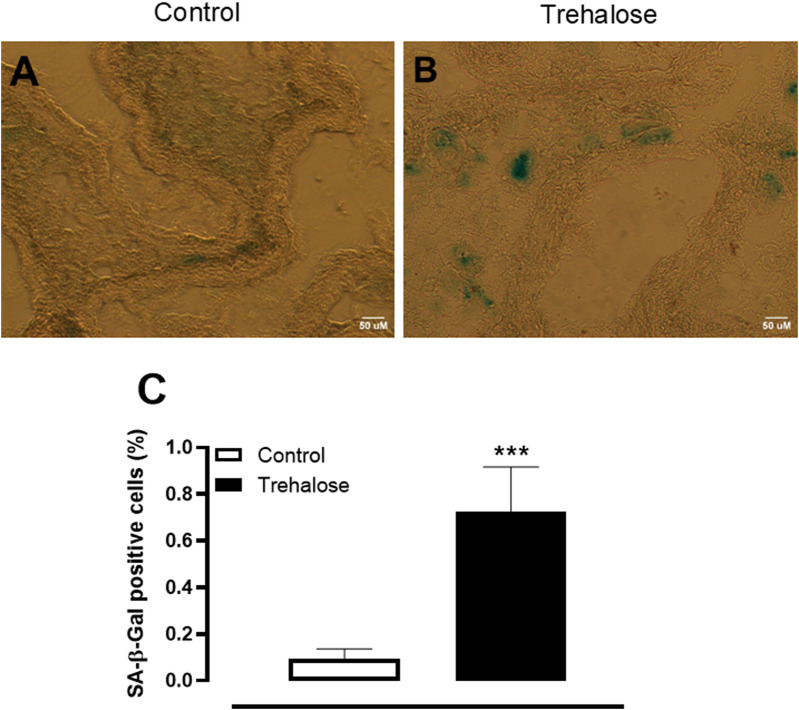
Representative images **(A, B)** and quantification **(C)** of senescent cells in bladder urothelium from control and trehalose-treated mice. Data represent the mean ± S.E.M. (n = 5). ***P < 0.001 compared with control group.

The cell senescence was evaluated in fresh-frozen bladder sections of control and trehalose-treated mice. As evidenced in Figure 4, trehalose treatment triggered a marked increase (approximately 7.8-fold; P < 0.001, Hedges’ *g* = 1.94) in senescence-associated β-galactosidase staining in urothelial layer of bladder tissue compared with control group.

### Western blot analysis for autophagy-related proteins

**FIGURE 5:**
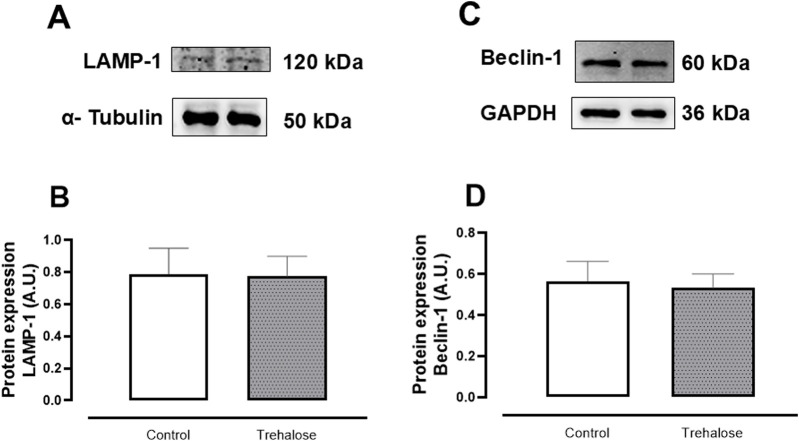
Representative images of Western blotting and densitometric ratios of LAMP-1 **(A, B)** and Beclin-1 **(C, D)** in bladder of control and trehalose-treated mice. Data represent the mean ± S.E.M. (n = 6).

Western blot analysis revealed that trehalose treatment was not capable of increasing LAMP1- (Figure 5A) and Beclin-1 (Figure 5B) protein expressions in bladder tissue compared with control group.

## Discussion

In the present study, we demonstrated for the first time that chronic treatment with trehalose led to bladder smooth muscle (BSM) hypercontractility, which was accompanied by increased ROS levels in the urothelium layer of the bladder in mice. Furthermore, *in vitro* ROS neutralization using pharmacological agents (apocynin and DPI) restored the increased BSM contraction, reinforcing the role of ROS in trehalose-induced BSM hypercontractility. Trehalose also induced cellular senescence in the bladder, likely as a consequence of increased ROS production. Interestingly, trehalose was not capable of increasing bladder weight, inducing hyperglycemia, or upregulating the expression of key autophagy proteins (LAMP-1 and Beclin-1) in the bladder, suggesting that the detrimental effects of this disaccharide on bladder tissue are independent of bladder remodeling, hyperglycemia, and the activation of the autophagy process.

Voiding problems related to bladder filling, storage, or emptying are complex and affect millions of people worldwide (Van Huele et al., 2024). The resulting signs and symptoms, collectively referred to as lower urinary tract symptoms (LUTS), include urinary urgency, frequency changes, and overactivity bladder (OAB), which may lead to urinary incontinence (Van Huele et al., 2024). OAB is a chronic condition characterized by neurogenic and/or myogenic impairments, with the latter commonly associated with smooth muscle dysfunctions (Calogero et al., 2018). The literature indicates that animal models of OAB exhibit increased BSM contractility and/or impaired relaxation (Mónica et al., 2008; Calmasini et al., 2017; Oliveira et al., 2022). In the present study, we showed for the first time that mice treated chronically with trehalose exhibited BSM hypercontractility, suggesting an OAB phenotype. Indeed, some studies in the literature have demonstrated impairments in smooth muscle reactivity induced by trehalose. For instance, male Wistar rats chronically treated with trehalose exhibited impaired endothelium-independent vascular relaxation and increased arterial stiffness in mesenteric arteries associated with oxidative and endoplasmic reticulum stress (McCarthy et al., 2019). These findings suggest that trehalose may play a detrimental role in smooth muscle of vascular and non-vascular tissues. Curiously, studies investigating the effects of trehalose on smooth muscle reactivity remain scarce, especially under physiological conditions. Therefore, variations in treatment duration and/or dosage could provide further insights into other potential effects of trehalose on bladder function.

Oxidative stress can be pragmatically defined as an imbalance in the production of ROS within a given system. The superoxide anion (O^2-^) is one of the most studied ROS primarily produced by the multi-component enzyme complex NADPH oxidase (Lassègue and Griendling, 2010). Considering that I) in the context of the lower urinary tract, increased ROS levels have been implicated in the pathophysiology of OAB in rodents (Alexandre et al., 2016; Calmasini et al., 2017; Silveira et al., 2024) and that II) trehalose treatment is able to increase oxidative stress in vascular tissues from Wistar rats (McCarthy et al., 2019), we investigated the role of ROS production in trehalose-induced BSM hypercontractility in mice. Our findings revealed a significant increase in O^2-^ levels in the urothelial layer, but not bladder smooth muscle, in trehalose-treated mice. Interestingly, the *in vitro* incubation with apocynin completely restored the trehalose-induced BSM hypercontractility. Although apocynin was initially classified as a selective NADPH oxidase inhibitor, subsequent studies have demonstrated additional pharmacological targets for this molecule, identifying it as a non-specific antioxidant (Savla et al., 2021). Therefore, in an attempt to further investigate the source of ROS, we used DPI, a more selective NADPH oxidase inhibitor (Reis et al., 2020). Similarly, DPI also restored trehalose-induced BSM hypercontractility. Albeit studies have shown that DPI also exhibits other pharmacological targets besides NADPH oxidase, such as the inhibition of xanthine oxidase (Reis et al., 2020), overall, our data strongly suggest a pivotal role for NADPH oxidase-derived O^2-^ in BSM hypercontractility induced by trehalose in mice.

One of the consequences of oxidative imbalance is cellular injury, which leads to the accumulation of senescent cells in tissues (Chen et al., 1995; Parrinello et al., 2003; Sun and Feinberg, 2021). Notably, cellular senescence driven by increased oxidative stress has been associated with voiding dysfunction (Ye et al., 2023; Wang et al., 2023), which prompted us to assess senescent cells in the bladders of trehalose-treated mice. We demonstrated that trehalose treatment was capable of increasing senescence-associated β-galactosidase activity in the bladder. This suggests that trehalose-induced BSM hypercontractility in mice may, at least in part, be associated with an increase in senescent cells, likely resulting from oxidative stress and ROS imbalance.

Finally, we assessed whether trehalose treatment was effective in activating the autophagy process in the bladders of mice. Interestingly, no changes were observed in the protein expression of Beclin-1 and LAMP-1, proteins responsible for phagophore formation and the fusion of the autophagosome with the lysosome, respectively (Rusmini et al., 2019; Debnath et al., 2023). This result suggests that BSM hypercontractility induced by trehalose is not associated with a supra-physiological regulation of the autophagy process. Similarly, McCarthy and colleagues demonstrated that chronic treatment with trehalose induced structural and biochemical changes (including increased O^2−^ levels) in the mesenteric artery of Wistar rats without altering the autophagy process (McCarthy et al., 2019).

Our study demonstrates that chronic treatment with trehalose induces BSM hypercontractility and increased ROS levels in the urothelium of mice, which may provide insights into the pathophysiology of human bladder dysfunction, particularly OAB. The ROS-associated mechanism, secondary to trehalose treatment, observed in our study suggests that oxidative stress could contribute to BSM hypercontractility in OAB, and targeting ROS might offer a potential therapeutic strategy. Accordingly, existing studies on ROS in both human and rodent bladder disorders support the relevance of our findings (Alexandre et al., 2016; Wang et al., 2023). However, clinical studies specifically addressing the effects of trehalose on human bladder function are lacking, which refrain us from translating the results to humans. Therefore, these insights could guide future clinical research on trehalose and ROS modulation in bladder disorders.

The present study also raised some open questions. One of them is the exact mechanism by which trehalose increases the levels of ROS independently of autophagy activation. Indeed, recent evidence has demonstrated that trehalose can exert effects independently of autophagy activation. For instance, Mizunoe et al. showed that trehalose can activate p62/SQSTM1 in an autophagy-independent manner (Mizunoe et al., 2018). The accumulation of p62 and inclusion bodies containing both ubiquitylated proteins and p62 have been identified in several human disorders (Kuusisto et al., 2001; Stumptner et al., 2002) and may be associated with mitochondrial dysfunction and increased levels of ROS. Of note, high levels of ROS may, in turn, promote cellular senescence through DNA damage which may further contribute to BSM hypercontractility. Whether this same signaling pathway also occurs in the bladder of mice remains unclear and may be an interesting mechanism to be explored in the future. Among the limitations of this study, it is worth mentioning that: *in vivo* assessments (for instance, voiding behavior) were not addressed, the study was not conducted in a blinded manner and a more in-depth exploration of urothelium-smooth muscle signaling in terms of contractility is needed. Future studies should be conducted to address these matters.

## Conclusion

Collectively, our data demonstrate for the first time that trehalose treatment induces BSM hypercontractility in mice, which is associated with increased levels of O^2−^ and senescence-associated β-galactosidase activity, and is independent of autophagy activation, bladder remodeling and hyperglycemia. These findings suggest that trehalose administration represents a valuable and effective model for studying OAB in mice, offering insights into its pathophysiology, particularly associated with oxidative stress and cellular senescence.

## Data Availability

The original contributions presented in the study are included in the article/Supplementary Material, further inquiries can be directed to the corresponding author.
