# Supplementary figures and images for: Trehalose induces bladder smooth muscle hypercontractility in mice: involvement of oxidative stress and cellular senescence
Source: Front Physiol. 2025 Apr 4;16:1572139. doi: 10.3389/fphys.2025.1572139 (PMC12006093; doi:10.3389/fphys.2025.1572139)

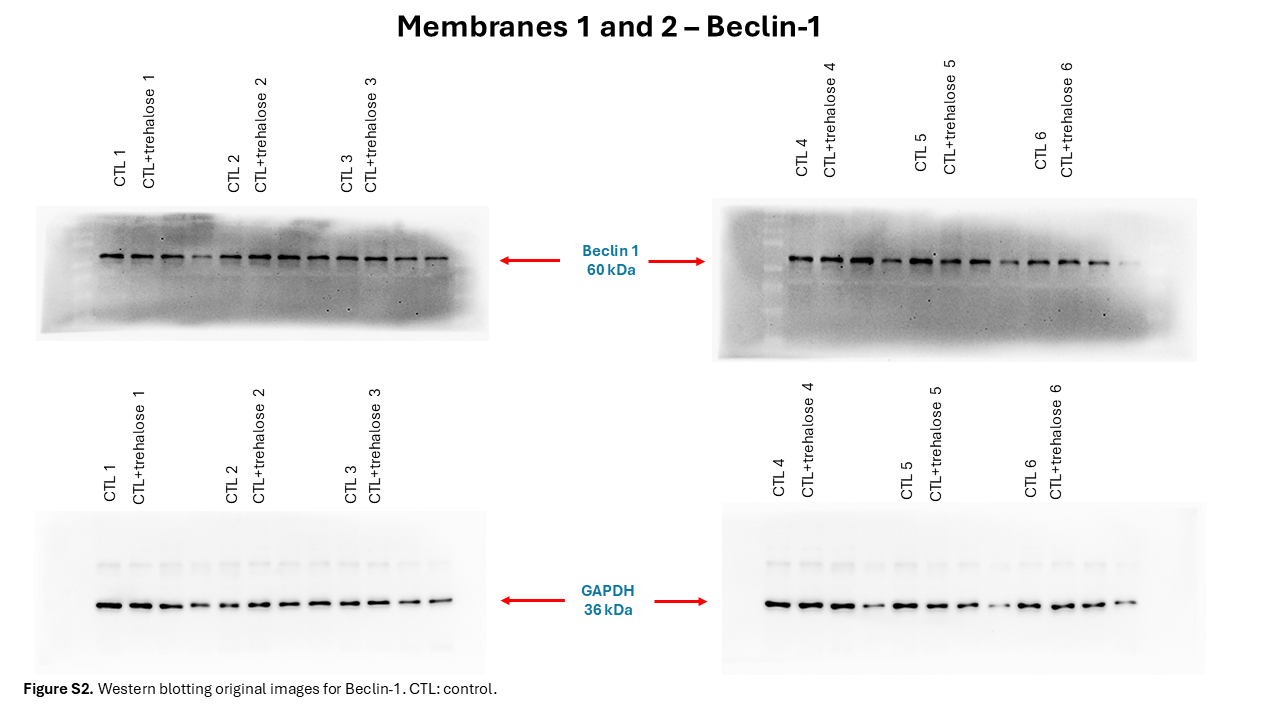

Supplement: Supplementary file 1 [file DataSheet1.zip › WB images 2.tif]

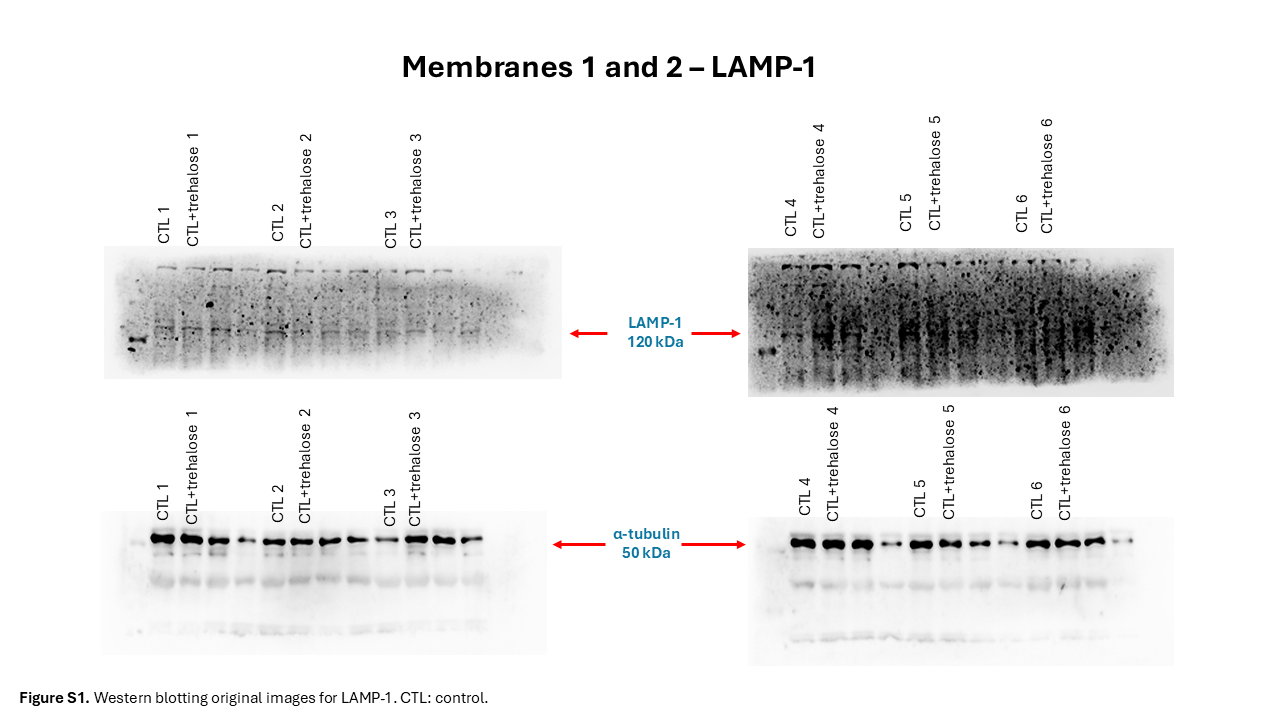

Supplement: Supplementary file 1 [file DataSheet1.zip › WB images 1.tif]
